# Large-scale frontoparietal theta, alpha, and beta phase synchronization: A set of EEG differential characteristics for freezing of gait in Parkinson’s disease?

**DOI:** 10.3389/fnagi.2022.988037

**Published:** 2022-10-26

**Authors:** Fatemeh Karimi, Quincy Almeida, Ning Jiang

**Affiliations:** ^1^Department of Systems Design Engineering, University of Waterloo, Waterloo, ON, Canada; ^2^Movement Disorders Research and Rehabilitation Consortium, Department of Kinesiology and Physical Education, Wilfrid Laurier University, Waterloo, ON, Canada; ^3^National Clinical Research Center for Geriatrics, West China Hospital, Sichuan University, Chengdu, China; ^4^Med-X Center for Manufacturing, Sichuan University, Chengdu, China

**Keywords:** Parkinson’s disease (PD), freezing of gait (FOG), EEG, brain oscillations, phase synchronization (PS), phase-locking value (PLV), Surface Laplacian (SL), dopamine

## Abstract

Freezing of gait (FOG) is a complex gait disturbance in Parkinson’s disease (PD), during which the patient is not able to effectively initiate gait or continue walking. The mystery of the FOG phenomenon is still unsolved. Recent studies have revealed abnormalities in cortical activities associated with FOG, which highlights the importance of cortical and cortical-subcortical network dysfunction in PD patients with FOG. In this paper, phase-locking value (PLV) of eight frequency sub-bands between 0.05 Hz and 35 Hz over frontal, motor, and parietal areas [during an ankle dorsiflexion (ADF) task] is used to investigate EEG phase synchronization. PLV was investigated over both superficial and deeper networks by analyzing EEG signals preprocessed with and without Surface Laplacian (SL) spatial filter. Four groups of participants were included: PD patients with severe FOG (*N* = 5, 5 males), PD patients with mild FOG (*N* = 7, 6 males), PD patients without FOG (*N* = 14, 13 males), and healthy age-matched controls (*N* = 13, 10 males). Fifteen trials were recorded from each participant. At superficial layers, frontoparietal theta phase synchrony was a unique feature present in PD with FOG groups. At deeper networks, significant dominance of interhemispheric frontoparietal alpha phase synchrony in PD with FOG, in contrast to beta phase synchrony in PD without FOG, was identified. Alpha phase synchrony was more distributed in PD with severe FOG, with higher levels of frontoparietal alpha phase synchrony. In addition to FOG-related abnormalities in PLV analysis, phase-amplitude coupling (PAC) analysis was also performed on frequency bands with PLV abnormalities. PAC analysis revealed abnormal coupling between theta and low beta frequency bands in PD with severe FOG at the superficial layers over frontal areas. At deeper networks, theta and alpha frequency bands show high PAC over parietal areas in PD with severe FOG. Alpha and low beta also presented PAC over frontal areas in PD groups with FOG. The results introduced significant phase synchrony differences between PD with and without FOG and provided important insight into a possible unified underlying mechanism for FOG. These results thus suggest that PLV and PAC can potentially be used as EEG-based biomarkers for FOG.

## Introduction

Freezing of gait (FOG), the sudden episodic inability to move the foot forward despite the intention to walk, is a debilitating symptom of Parkinson’s Disease (PD) (Giladi and Nieuwboer, 2008; Browner and Giladi, 2010). The pathophysiological mechanism of the FOG phenomenon has not been understood due to its complexity and episodic nature (Nieuwboer and Giladi, 2013; Gao et al., 2020; Marquez et al., 2020). While dopaminergic disorders of the basal ganglia (BG) are the core of PD, numerous studies have illuminated the multiplicity of brain structures and patterns of cortical activities affected by PD and FOG as well as its severity (Li et al., 2020; Marquez et al., 2020; Weiss et al., 2020; Jin et al., 2021). The majority of the current hypotheses in the literature on the underlying mechanisms of FOG suggest some level of dysfunction in the cortical structures such as supplementary motor area (SMA) and motor cortex, as well as the communication between these regions and BG (Iansek et al., 2006; Iansek and Danoudis, 2017; Marquez et al., 2020). Cortical activity plays a crucial role in stabilized gait control, especially during challenging tasks such as obstacle crossing, changes in speed, and dual tasks, which are all FOG-provoking situations emphasizing on the significance of cortical networks involvement in FOG (Delval et al., 2020). It is therefore highly likely that the mechanism of FOG involves higher-level cortical modulators from non-motor perspective (e.g., cognitive and attention-related networks) rather than only the motor perspective (e.g., prefrontal cortex (PFC), SMA, premotor cortex, and motor cortex) (Li et al., 2020; Weiss et al., 2020). On the other hand, regarding the role of midbrain dopamine, dopaminergic system dynamics have been suggested to be the main contributors to whole-brain coordination through synchronicity and time perception (Nantel et al., 2012; Marinho et al., 2018; Beeler and Dreyer, 2019). Finding the links across cortical oscillations that interact with subcortical regions and might be affected by dopamine loss in PD, are thus essential in addressing current issues in deep understanding of the FOG phenomenon. The existing body of research reports abnormalities in the power spectrum and amplitude of various cortical oscillations, including movement-related cortical potentials (MRCP), theta (4–8 Hz), alpha (8–12 Hz), low beta (12–21 Hz), and high beta (21–35 Hz) bands associated with FOG. Although there is a growing body of literature that recognizes the relationship between brain oscillations, gait and FOG (Wang and Choi, 2020), the field is still far from providing a systematic understanding of how brain oscillation dynamics contribute to pathological gait such as FOG.

BG plays a central role in timing and sequencing through distributed, parallel neuronal networks to connect and integrate functions (Flash et al., 1992; Lewis and Barker, 2009; Nantel et al., 2012; Marinho et al., 2018; Beeler and Dreyer, 2019). The footprint of timing dysfunction is traceable in several theories about FOG, such as motor breakdown as a result of motor deficits accumulation over time, conflict-resolution deficit, especially during time-constraint tasks, and overload of information processing capacity in motor, sensory, cognitive, and limbic inputs to BG due to insufficient dopaminergic cells in a limited time window. Considering the importance of time in underlying neurocomputational mechanisms (Cohen, 2011); and the independency of phase and power as dimensions of information, in this study, we take one step beyond to investigate what modulates the amplitude and power of the cortical oscillation: phase. The amplitude of higher frequency bands is controlled by the phase of the lower frequency bands (Buzsáki, 2009). Therefore, the investigation of the phase of the lower frequency bands, especially at a large scale, is crucial in the exploration of the underlying mechanisms of FOG. Although the number of studies on the phase features related to gait and FOG is limited (Arnulfo et al., 2018), in a recent study, high beta-gamma PAC in the primary motor cortex was reported as a FOG-associated feature (Yin et al., 2022).

In the current study, we investigated the phase-locking value (PLV) of distributed cortical areas of the locomotor network (e.g., frontoparietal, SMA, and primary motor area) as well as higher-level cortical modulators (e.g., PFC) to explore neural networks associated with FOG. The PLV was explored in slow cortical potentials and low frequencies such as theta and alpha, as well as movement-related beta oscillation during a simple lower-limb movement task. Furthermore, although phase synchrony is considered to be the mechanism for neural group communication across both close and distant brain areas, phase-amplitude coupling (PAC) is mainly considered as the mechanism to control long-distance communication based on the fact that slow oscillations can propagate at larger scales compared to fast oscillations (Munia and Aviyente, 2019). As a result, in this study, PAC of the frequency bands was explored to investigate the possible relevance of the excessive beta power and the phase of lower frequencies in PD patients with FOG. In addition, considering the involvement of the multilayer neocortex and subcortical regions of the brain in FOG, phase features have been investigated with and without a spatial filter, Surface Laplacian (SL), that improves the spatial resolution of EEG signals and provides complementary information (Kayser and Tenke, 2015). Prior studies about the origin of freezing in PD have been suggested to involve spatiotemporal disorder as a core motor problem which underlies freezing (Vercruysse et al., 2012). In a broader context, there is likely a universal mechanism and upstream cause underlying the phenomenon (Shine et al., 2011). So, investigating phase features of brain oscillations correlated with FOG might help converge recent findings in a meaningful way to help identify unified mechanisms in FOG. This study offers a better understanding of possible underlying mechanisms for FOG, as well as biomarkers to distinguish PD patients with and without FOG based on EEG phase features. The results from this study can also help provide insight into new treatment paths to rehabilitate FOG.

## Materials and methods

### Participants

Forty-one participants including 14 PD patients without FOG, 14 PD patients with FOG and 13 age-matched healthy control participants (PD without FOG: mean age = 77 years, range = 65–87 years, three females; PD with FOG: mean age = 74 years, range = 63–90 years, one female; healthy controls (HC): mean age = 77 years, range = 68–89 years, three females) took part in the experiment. The PD patients were recruited from the Movement Disorders Research and Rehabilitation Center (MDRC) at the Wilfrid Laurier University (Waterloo, Ontario). Participants with any head trauma, neurological disorder, severe vision or hearing problems and severe movement control limitations such as dyskinesia were excluded. All patients were in their optimally medicated state to avoid the confound of exacerbated motor symptoms. The severity of patients’ motor symptoms was assessed based on the Unified Parkinson’s Disease Rating Scale (UPDRS). PD patients with FOG were identified by the answer to question 14 in MDS-UPDRS-III (motor subsection), which confirms the presence of FOG. In addition, an experienced clinician reconfirmed the occurrence of FOG before each experiment session, according to the standardized protocol (Almeida and Lebold, 2010). The procedure involved a modified Timed Up and Go test where the participant would have started from a seated position, raised themselves out of a chair with arms across their chest, walked ∼3 m but through a doorway into an adjacent clinic room that was cluttered with other desks and chairs, then returned to in front of their chair where they completed degree turns in both the left and right directions, before sitting back down. PD patients with FOG were divided into two subgroups of PD with mild and severe FOG. PD + sFOG were defined as those who experienced observable FOG episodes whenever walking or turning that severely affect their daily activities and independence, while PD + mFOG were defined as those who experienced FOG occasionally when provoked only during more complex tasks such as turning (based on patient history). In addition, the participants were instructed to perform 20 trials of videotaped walking tasks on a 10-m walkway. Participants were asked to walk after hearing an auditory “go” cue. PD + FOG who experienced FOG episodes longer than 3 s during turning or normal walking were considered PD + sFOG. The videotaped walking tasks were used to determine the dominant foot for each participant.

HC were recruited from The Waterloo Research in Aging Participant pool at the University of Waterloo. The sample size was determined by availability of PD patients. The study was approved by the Research Ethics Board at the University of Waterloo and Wilfrid Laurier University. A written informed consent form was obtained from each participant prior to the experiment, according to the Declaration of Helsinki.

In the current study, patients were confirmed to have idiopathic Parkinson’s. To maximize the number of participants experiencing FOG, any participants that met all the inclusion criteria for the study and were confirmed to experience FOG were recruited first. Subsequently, healthy controls and PD without FOG were recruited to match for age [*F*(2, 39) = 1.1, *P* = 0.3], severity using the UPDRS, levodopa equivalent dose (LED), and disease duration (*P* = 0.4, *P* = 0.2, and *P* = 0.4, respectively). PD with mild and severe FOG groups did not differ in age (*P* = 0.4), severity of motor symptoms (UPDRS-III: *P* = 0.4), LED (*P* = 0.07), disease duration (*P* = 0.3). Individual participant details of all groups can be found in our previous paper (Karimi et al., 2021). Table 1 represents the participant demographics and clinical characteristics. Further details of our experimental setup can be found in our previous paper (Karimi et al., 2021).

**TABLE 1:** Mean ± standard deviations for participant demographics and clinical characteristics.

	HC	PD − FOG	PD + mFOG	PD + sFOG
N (male/female)	13 (10/3)	14 (11/3)	7 (6/1)	5 (5/0)
Age (year)	77.61 ± 5.65	74.5 ± 6.67	76.7 ± 7.5	79.6 ± 1.14
Disease duration (year)	N/A	8.07 ± 5.18	11 ± 5.74	7.8 ± 5.11
UPDRS-III	N/A	28.66 ± 7.16	34.21 ± 12.7	28 ± 6.85
LED (mg/day)	N/A	449.42 ± 358.59	585.8 ± 265.7	969 ± 468.03

UPDRS-III, Unified Parkinson’s Disease Rating Scale–III; LED, Levodopa Equivalent Dose.

### EEG and EMG recordings

EEG data were recorded using a 32-channel wireless EEG system (g.Nautilus, Guger Technologies, Austria). EEG signals were sampled at a sampling rate of 250 Hz. EEG data were collected from 17 channels following 10–20 international standard positions: Fp1, Fp2, Af3, Af4, F3, Fz, F4, Fc1, Fc2, C3, Cz, C4, Cp1, Cp2, P3, Pz, and P4. The reference electrode was placed on the right ear lobe.

For all individuals, the EMG was acquired using an 8-channel TELEMYO 2400 system (NORAXON INC.). Four wireless EMG sensors with a sampling frequency of 1,000 Hz were placed on the tibialis anterior (TA) and soleus muscles (SOL) on both legs.

### Experimental procedures

All participants were invited to the MDRC for the experimental sessions. For PD patients, the respective clinical assessment was performed within 2 weeks of the experimental session. During the experiment, participants were instructed to perform ankle dorsiflexion (ADF) (e.g., lifting the toe) to the maximum possible contraction with the dominant foot, while sitting in a comfortable chair with their arms rested on armrests. To minimize eyes or head movements and reduce the cognitive load unrelated to the cues, they were asked to look at the center of a black “ + ” sign on a white background. One session with 15 trials was recorded for each participant, with an interval of 15 s between every two trials. Participants were expected to prepare for the task when they heard “ready” and execute ADF when they heard the “go” cue. The “ready” and “go” auditory cues, with a 2 s interval, were played for each trial through a speaker with a computer-generated voice.

### Data processing

EEG and EMG data were analyzed offline after the experiment session using a customized MATLAB function (Mathworks, USA R2020a). EMG signals recorded from the TA muscles of the dominant foot (EMG -TA) were used to identify onset timings of the ADF. EMG was initially filtered using a second-order Butterworth band-pass filter with the bandwidth between 20 and 120 Hz, and then down-sampled to 250 Hz to maintain consistency with that of the EEG data. To enhance the detection accuracy of the movement onset, the Teager–Kaiser Energy Operator (TKEO) was applied to the EMG data (Solnik et al., 2010). By being independent of the initial phases, TKEO algorithm instantly responses to the abrupt temporal changes in the EMG signal while improving the SNR and minimizing erroneous EMG burst detection (Solnik et al., 2010). Finally, a threshold value was manually selected for each subject to determine the movement onset. The EEG data was initially band-pass filtered by a third-order Butterworth filter between 0.05 Hz and 50 Hz. The filtered EEG data were processed by independent component analysis (ICA) using the EEGLAB toolbox. Source components containing eye blinks, severe head motion or EMG artifacts were removed (Delorme and Makeig, 2004). The phase and amplitude features are investigated both with and without SL (López-Larraz et al., 2014; Xu et al., 2014). More details about the implementation of SL are provided in the next section.

**FIGURE 1:**
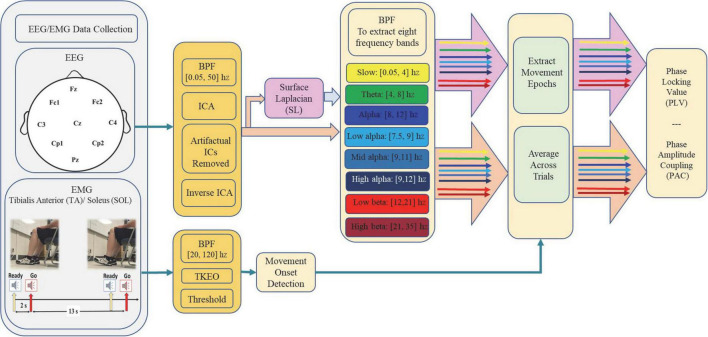
Schematic representation of experimental setup and data processing.

The outliers were discarded from further analysis based on the excessive muscle activities in leg muscles prior to the “Go” auditory cue, motion, or other types of artifacts. Excessive muscle activities were identified when EMG amplitude was above the movement onset detection threshold in the non-dominant foot over the time interval [−3, 5] s with respect to the auditory “Go” cue and/or in case EMG amplitudes from the dominant foot was above the movement onset detection threshold over the same time interval except for the time course of ADF execution ([0, 5] s with respect to the auditory “Go” cue). In general, the 5–15 most consistent trials from each participant were manually selected, with a total number of 122 ± 2 trials in each group. For those participants for whom their left leg was dominant, the EEG channels on the left and right sides were switched during the analysis. Figure 1 represents schematic for experimental setup and data processing in this study.

### EEG data processing

#### Surface laplacian

SL represents the second spatial derivative of the instantaneous spatial voltage distribution, which suppresses the signals with low spatial frequency (i.e., signals originating from distributed and/or deep generator sources) (Kayser and Tenke, 2015). As a result, applying SL emphasizes on superficial, radial sources cortical generators and attenuate deeper sources, vertical connections, and broadly distributed generators (Lu et al., 2013; Cohen, 2019). While SL reduces the effect of volume conduction, the effect of this spatial filter on phase-sensitive measures such as PLV is still controversial (Tenke and Kayser, 2015; Jian et al., 2017a,b). Therefore, in this study, data has been investigated both with and without applying SL to the EEG data. In order to implement SL, the estimate of the second derivative of the scalp voltage based on a finite-difference method was used (Hjorth, 1975). SL was applied on Fz, Cz, Fc1, Fc2, Cp1, and Cp2 by subtracting the averaged signal of the four surrounding orthogonal electrodes from the center electrode.

#### Phase locking value

The communication between pre and post-synaptic neurons takes place *via* phase synchronization, both for short distances within a brain region up to long ranges between distant brain areas. PLV is a measure that represents the level of neural phase synchrony and EEG connectivity at a specific frequency range between two neural groups (Jian et al., 2017a). In other words, neural groups that oscillate at the same frequency are phase-locked to each other (Zazio et al., 2021). In this paper, PLV is calculated according to Aydore et al. (2013):


(1) $$PLV(n)=\frac{1}{N}\left|\sum_{k=1}^{N}e^{i(\phi_{\left(n,k\right)}-\varphi_{\left(n,k\right)})}\right|$$

where *N* is the number of trials, ∅_(*n,k*)_ and φ_(*n,k*)_ are instantaneous phase of two different electrodes computed by Hilbert Transformation. The magnitude of the PLV, i.e., how much two oscillations are phase-locked to each other, thus quantifies effective interactions between neural groups. PLVs were calculated for the time interval [−5, 5] s, with respect to movement onset, thus including movement preparation, initialization and execution as well as time samples at rest. As the focus of this research is to investigate phase synchronization related to movement preparation that results in successful movement execution, two steps were taken: Firstly, PLV traces that represent significant PLVs outside [−3, 2] s were not reported as they were not task-relevant. Secondly, considering that the “ready” auditory cure occurred at time −2 s, the transient significant PLVs between [−3, −1] s, were not reported as significant PLVs related to movement preparation. This step was taken to limit the effect of the phase synchronizations related to “ready” auditory cue and focus on the phase synchronizations that lead to movement execution. Lastly, as time zero represents movement onset, the PLV traces that represent significance only after 1 s were not reported as significant PLVs related to movement preparation and initiation. PLVs were also baseline-corrected to the mean of the pre-stimulus period, [−10, −3] s with respect to the movement onset.

#### Time-frequency phase amplitude coupling

The coupling between the phase of slow oscillations and the amplitude of fast oscillations, referred to as PAC, is one of the mechanisms underlying neural binding. To assess PAC between frequency bands with abnormal activities among different groups, a novel robust time-frequency-based PAC measure was implemented (Munia and Aviyente, 2019). The method is based on a complex time-frequency distribution, named Reduced Interference Distribution (RID)-Rihaczek distribution and mean vector length (MVL). In this paper, we specifically investigated coupling between phase of slower cortical potentials ([0.5, 7.5] Hz) with the amplitude of alpha and beta frequency bands. PAC features were calculated for the two time intervals [−3, 1] s and [−3, 2] s, with respect to movement onset, thus including movement preparation, initialization and execution. Only PAC plots that represented highly distributed differences across groups over investigated frequencies were reported in comodulogram to show the coupling between high and low frequency.

### Statistical analysis

To determine statistical significance of PLV at different time points, Raileigh’s *Z* score for non-uniformity of circular data was used as following (Fisher, 1993; Shakeel et al., 2020):


(2) $$Z=N{\left(PLV\right)}^{2}$$

The *p*-value of Rayleigh’s test (function *circ_rtes* in Matlab) was calculated for each time point in the interval using CircStats toolbox (Berens, 2009). In the test, the null hypothesis is that the population is uniformly distributed around the circle with the alternative hypothesis that the population is not uniformly distributed, but rather has a specified mean angle. Unless specified otherwise, a significance level of 0.05 was used throughout the analysis. Correction for the false discovery rate (FDR) was also conducted using Benjamini and Hochberg test. All statistical analysis was performed [Matlab R2021a (the Mathworks, Inc.)].

## Results

**FIGURE 2:**
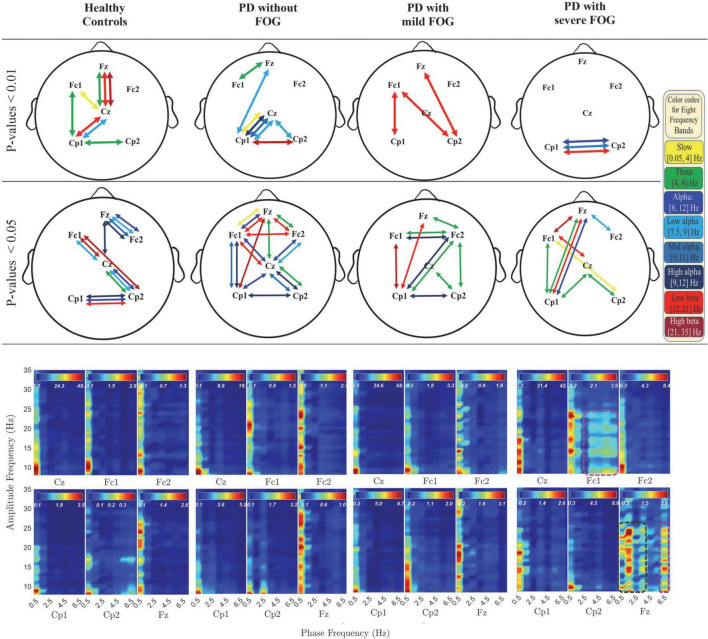
Significant PLV between different channels over different frequency bands (top two rows) and PAC coupling between lower frequency bands (slow cortical potentials and theta) and higher frequency bands (alpha and beta frequency bands) (bottom row) with SL in all groups. In the top rows, different colors represent different frequency bands: Yellow: slow cortical potentials; green: theta; navy blue: alpha; light blue: low alpha; blue: middle alpha; dark blue: high alpha, light red: low beta; dark red: high beta. First and second row represent significant PLVs for *p* < 0.01 and *p* < 0.05, FDR-corrected, respectively. In the lower row, PAC between lower frequency bands (slow cortical potentials and theta) and higher frequency bands (alpha and beta frequency bands) with SL are presented over [–3, 1] s for all groups. Dashed pink lines represent the coupling of the phase of theta with alpha and low beta frequency bands. Dark green dashed lines represent the PAC between higher slow cortical potentials and alpha and beta in Fz.

**FIGURE 3:**
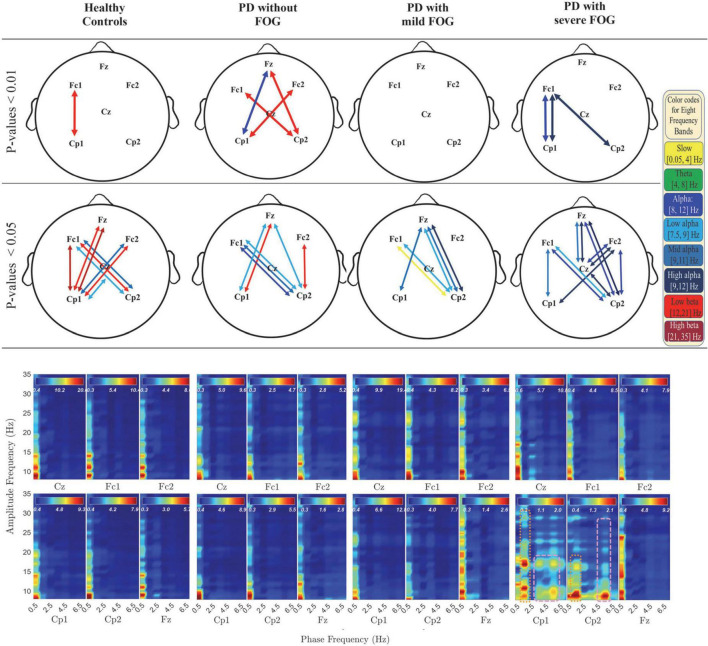
Significant PLV between different channels over different frequency bands (top two rows) and PAC coupling between lower frequency bands (slow cortical potentials and theta) and higher frequency bands (alpha and beta frequency bands) (bottom row) without SL in all groups. In the top row, different colors represent different frequency bands: Yellow: slow cortical potentials; green: theta; navy blue: alpha; light blue: low alpha; blue: middle alpha; dark blue: high alpha, light red: low beta; dark red: high beta. First and second row represent significant PLVs for *p* < 0.01 and *p* < 0.05, FDR-corrected, respectively. In the lower row, PAC between lower frequency bands (slow cortical potentials and theta) and higher frequency bands (alpha and beta frequency bands) without SL over [–3, 1] s is presented for all groups. Dashed pink lines represent the coupling of the phase of theta with alpha and beta frequency bands. Pink dotted lines represent the PAC between higher slow cortical potentials and alpha and beta.

In Figures 2, 3, the phase-locked channels with two levels of significant PLVs are presented (top two rows) with and without SL for all groups: Healthy Control participants (HC), PD without FOG (PD-FOG), PD with mild FOG (PD + mFOG), and PD with severe FOG (PD + sFOG). Top row represent a significance level of 0.01, and lower row represent PLVs with significance level of 0.05. Different colors represent significant PLV in different frequency bands, including yellow: slow cortical potentials; green: theta; navy blue: alpha; light blue: low alpha; blue: middle alpha; dark blue: high alpha; light red: low beta; dark red: high beta. Significant PLVs (over [−1, 1] s with respect to the movement onset) are presented to determine phase-locked channels during movement preparation and execution before and after the auditory “go” cue. In the following, differences between groups are discussed in detail at two different spatial resolutions.

### Radial superficial connections (with SL)

The analyses of PLV and PAC at radial and superficial networks (with SL) for different frequency bands are shown in Figure 2. The results revealed distinctions between PD without and with FOG groups as well as PD + mFOG and PD + sFOG, especially over theta frequency band.

### Primary motor area (Cz) centrally phased locked to frontal and parietal areas in HC and PD-FOG

As seen in the top rows of Figure 2, a common observable feature between HC and PD-FOG is that Cz is phase-locked to Fz, over theta. Plus, Cz is phased locked to Cp1, and Cp2 over low alpha frequency band. This pattern is missing in PD + FOG groups. In addition, HC represents a strong phase synchronization (*p* < 0.01) between the prefrontal area (Fz) and primary motor cortex (Cz) in theta and beta frequency bands, but no such synchronization can be seen in PD-FOG.

#### Theta between frontal areas and Cz is missing in PD + FOG

At the superficial networks, the most distributed phase synchronization is observable in the theta frequency band in all groups. HC and PD-FOG show phase synchronization between Fz and Cz. By contrast, this feature is missing in both PD groups with mild and severe FOG. In the two PD + FOG groups, unlike HC and PD-FOG, the theta phase synchrony is higher in parietal areas rather than (pre-)frontal areas and presents frontoparietal phase synchronization. More importantly, this shift in theta phase synchrony increases with the severity of FOG. While in PD + mFOG theta synchrony is observable in the (pre-) frontal areas along with large-scale phase synchrony between Cp1 and Fc2, in PD + sFOG there is no theta phase synchrony in the frontal areas. In PD + sFOG, theta phase synchrony is more pronounced in the parietal areas with dominance on the left hemisphere. While in PD + mFOG, there is interhemispheric frontoparietal theta phase synchrony between Cp1 and Fc2, PD + sFOG represents one large-scale theta phase synchrony between Cp1 and Fz. These results suggest that there is a direct relationship between large-scale interhemispheric frontoparietal theta phase synchrony at the superficial networks and FOG. The shift of this large-scale theta phase synchrony from the phase-locking between left parietal areas (Cp1) and right SMA (Fc2) in PD + mFOG to phase-locking between left parietal areas (Cp1) and prefrontal areas (Fz) in PD + sFOG, might represent the relationship between this feature and the severity of FOG.

#### Lack of low alpha phase synchrony between Cp1 and Cz in PD + FOG

In Figure 2, significantly high low beta phase synchrony (*p* < 0.01) is observable between Cp1 and Cz in HC and PD-FOG. In these groups, the same pattern is observable between Cp2 and Cz, with lower significance in HC. In contrast, the low alpha phase synchrony between parietal areas (CP1 and Cp2) and Cz are missing in the two PD + FOG groups. PD + sFOG represents only low alpha phase synchrony in the frontal areas between Fz and Fc2, and PD + mFOG does not represent any low alpha phase synchrony at superficial layers. However, the groups represent large-scale frontoparietal low alpha phase synchrony at deeper networks.

#### Lack of high alpha phase synchronization between bilateral parietal areas in PD + sFOG

Bilateral parietal high alpha phase synchrony between Cp1 and Cp2 is present in all groups except PD + sFOG, where a highly significant (*p* < 0.01) phase synchrony is seen between Cp1 and Cp2 in low alpha, alpha, and low beta, as observable in the top row in Figure 2. On the other hand, PD + mFOG represents high alpha phase synchrony at the same significance level (*p* < 0.05) as HC and PD-FOG. Interestingly, phase synchrony in mid alpha is an exclusive phase synchrony feature of PD + sFOG between CP1 and Cp2, which is absent in all other groups.

#### Phase of frontal delta and theta is coupled with the amplitude of low beta in PD + sFOG

Figure 2 (lower row) presents the comodulogram for PAC values between lower frequency bands ([0.5, 7.5] Hz) and higher investigated frequency bands (alpha and beta) for all channels and all groups over the movement preparation and execution [−3, 1] s with respect to the movement onset. The most striking feature is the abnormal PAC of lower frequencies and theta with the amplitude of alpha and beta frequency bands in PD + sFOG group (dashed dark green and pink lines) in Fz and Fc1. The result suggests that, unlike all other groups, in PD + sFOG, the phase of theta frequency band is coupled with the amplitude of alpha and low beta in Fc1 and Fz. In all other groups, the amplitude of alpha and beta is coupled with the phase of slow cortical potentials (less than 1.5 Hz). More importantly, in PD + sFOG, the phase of the lowest frequencies is not coupled with the amplitude of alpha and beta. Table 2 represents *p*-values for electrode pairs with significant phase synchrony when SL is utilized.

**TABLE 2:** Pairs of electrodes with significant PLVs over different frequency bands with SL for all groups (*p* < 0.05).

Group	SCP	Theta	Alpha	Low alpha	Mid alpha	High alpha	Low beta	High beta
HC	Fc1-Cz (0.003)*	Cz-Cp2 (0.044) Cz-Fz (0.0073)* Cp1-Cp2 (0.0007)* Fc1-Cp1 (0.002)*	Fz-Fc2 (0.027) Cz-Cp2 (0.044) Cp1-Cp2 (0.019)	Fc1-Cz (0.01) Cz-Cp1 (0.00011)* Fz-Fc2 (0.022) Cz-Cp2 (0.017)	Fz-Fc2 (0.047)	Fz-Cz (0.01) Fz-Fc2 (0.032) Cp1-Cp2 (0.022)	Cz-Fz (0.007)* Cz-Cp1 (0.0013)* Fc1-Cp1 (0.015) Cp1-Cp2 (0.032)	Cz-Fz (0.00008)* Cz-Fc1 (0.01) Fc1-Cp2 (0.03)
PD-FOG	Cz-Cp1 (0.0012)*	Fz-Fc1 (0.0023)* Fz-Fc2 (0.041) Fz-Cz (0.013) Cz-Cp2 (0.036)	Fz-Fc1 (0.022) Cz-Fc1 (0.028) Cz-Fc2 (0.026) Cz-Cp1 (0.025)	Cz-Fc1 (0.03) Fz-Cp1 (0.0047)* Cz-Cp1 (0.0037)* Cz-Cp2 (0.0062)*	Cz-Cp1 (0.0037)* Cz-Cp2 (0.041) Fc1-Cp1 (0.029)	Cz-Cp1 (0.0016)* Cz-Cp2 (0.038) Fc1-Cp1 (0.034) Cp1-Cp1 (0.027)	Fz-Fc1 (0.015) Fz-Fc2 (0.034) Cz-Fc1 (0.034) Fc1-Fc2 (0.018)	Fz-Cp1 (0.041) Cp1-Cp2 (0.0038)*
PD + mFOG	−	Fz-Fc2 (0.04) Fc1-Fc2 (0.036) Fc2-Cp1 (0.045) Fc2-Cp2 (0.023) Cz- Cp2 (0.044)	−	−	−	Fc1-Fc2 (0.017) Fc2-Cp1 (0.016) Cp1-Cp2 (0.028)	Fc1-Cp1 (0.0012)* Fc1-CP2 (0.005)* Cp2-Fz (0.0032)* Fz-Cp1 (0.037)	Fc1-Cp1 (0.026)
PD + sFOG	Fc1-Cp2 (0.037)	Fc1-Cp1 (0.025) Cz-Cp1 (0.04) Cz-Cp2 (0.04)	Cp1-Cp2 (0.008)*	Fz-Fc2 (0.049)	Cp1-Cp2 (0.007)*	−	Fc1-Cz (0.013) Fz-Cp1 (0.015) Cp1-Cp2 (0.006)*	Fz-Fc1 (0.027)

*p < 0.01. Electrode pairs with PLVs with lower significance levels (*p* < 0.01) are presented in bold.

### Deeper neural networks (without SL)

PLVs without SL for different frequency bands are presented in Figure 3 (top two rows) with multicolor lines. These activities mainly represent large-scale crosstalk between the two hemispheres as well as frontoparietal phase synchrony in deeper networks. In addition, the phase synchrony is restricted to alpha and beta, and in one case, slow potentials.

#### Lack of low beta phase synchrony in PD with FOG

From the results represented in Figure 3 (top rows), it is observable that while HC and PD-FOG groups have significant frontoparietal inter-hemispheric phase synchrony in low beta frequency band, PD groups with FOG surprisingly do not show any level of phase synchrony in this frequency range.

#### Frontoparietal alpha band phase synchrony in PD + FOG represents right-hemisphere dominance

In Figure 3 (top rows), both PD + FOG groups represent high levels of alpha phase synchronization at deeper networks, especially in PD + sFOG. Interestingly, in PD + sFOG, alpha is the only frequency band that shows phase synchrony, with the greatest number of connections in the right parietal area. Highest levels of phase synchrony (*p* < 0.01) are observable between Fc1-Cp1 and Fc1-Cp2. Moreover, alpha frequency band represents high levels of phase synchrony in Fc1-Cp1. Besides the connection between parietal channels and Fc1, significant PLVs are also observable between Fz-Cz over mid alpha and high alpha. Importantly, PD + mFOG shares PLV similarity with PD + sFOG between Cp2-Fz over high alpha and Cp2-Fc1 over low alpha. However, PD + mFOG shows lower levels of phase synchrony compared to PD + sFOG. Similar to PD + FOG, in PD-FOG, phase synchrony between parietal areas and Fz over alpha sub-bands is observable, which is missing in HC.

#### Fz and Cz are phased locked through two alpha frequency sub-bands, in PD + sFOG

The only connection between Fz and Cz, at both superficial and deeper networks, in PD + sFOG is observable in mid alpha and high alpha when no SL is applied. This feature is exclusive to PD + sFOG, meaning that PD + mFOG does not show any significant level of phase synchrony between Fz and Cz, both with and without applying SL. On the other hand, the phase synchrony between Fz and Cz in HC and PD-FOG is observable in more superficial layers with theta phase synchrony as the common feature between both groups without FOG.

#### Frontoparietal low beta band phase synchrony in PD-FOG represents right-hemisphere dominance

In PD-FOG, frontoparietal interhemispheric low beta phase synchrony was highly significant (*p* < 0.01), which is an exclusive feature of this group. The phase synchrony in low beta frequency bands connects Cp1 to Fc2, and more interestingly, Cp2 to Fz and Fc1 in PD-FOG. As shown in Figure 3, the phase synchrony between Cp2 and Fz is only observable in PD patients, with a difference in frequency bands. PD-FOG represents large-scale frontoparietal phase synchrony in low beta, while PD + FOG represents phase synchrony mainly in the alpha frequency sub-bands between Cp2 and Fz. It should be noted that, like PD-FOG, frontoparietal interhemispheric low beta phase synchrony (Fc1-Cp2 and Fc2-Cp1) was also observable in HC but with lower significance (*p* < 0.05). Table 3 represents all *p*-values for electrode pairs with significant phase synchrony.

**TABLE 3:** Pairs of electrodes with significant PLVs over different frequency bands without SL for all groups (*p* < 0.05).

Group	SCP	Theta	Alpha	Low alpha	Mid alpha	High alpha	Low beta	High beta
HC	−	−	−	Cz-Cp1 (0.047) Fc1-Cp2 (0.04)	Fc1-Cp2 (0.031) Fc2-Cp1 (0.031)	−	Fz-Cp1 (0.046) Fc1-Cp1 (0.0074)* Fc1-Cp2 (0.016) Fc1-Cp1 (0.027)	Fc1-Cp1 (0.024) Fz-Cp1 (0.013)
PD-FOG	Fz-Fc1 (0.042)	−	Fz-Cp1 (0.0008)* Fc1-Cp2 (0.021)	Fz-Cp1 (0.033) Fc1-Cp2 (0.031) Fz-Cp2 (0.026)	Fc1-Cp2 (0.025)	−	Fz-Cp2 (0.0032)* Fz-Cp1 (0.011) Fc1-Cp2 (0.0051)* Fc2-Cp1 (0.0098)* Fc2-Cp2 (0.01) Cz-Fc1 (0.018)	−
PD + mFOG	Fc1-Cp2 (0.05)	−	Fz-Cp2 (0.036)	Fc1-Cp2 (0.031)	Fz-Cp1 (0.028) Fz-Cp2 (0.03)	Fz-Cp2 (0.035)	−	−
PD + sFOG	−	−	Fc1-Cp1 (0.0093)* Cz-Fc2 (0.018) Fc2-Cp2 (0.036) Fc1-Cp2 (0.021) Fz-Cp2 (0.035)	Fc1-Cp2 (0.049)	Fz-Cz (0.032) Fc1-Cp1 (0.04)	Fz-Cz (0.03) Cz-Fc2 (0.02) Fz-Cp2 (0.027) Cz-Cp2 (0.046) Fc1-Cp2 (0.0094)* Fc2-Cp1 (0.02) Fc1-Cp1 (0.0046)*	−	−

*p < 0.01. Electrode pairs with PLVs with lower significance levels (*p* < 0.01) are presented in bold.

#### Phase of high alpha is coupled with the amplitude of low beta in PD with FOG

**FIGURE 4:**
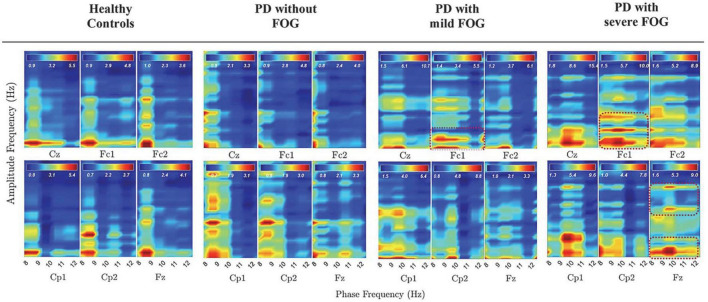
PAC between alpha and beta frequency bands without SL over [–3, 2] s in all groups. Dashed red lines represent PAC between all alpha frequency sub bands and low beta and high beta in PD with FOG.

Figure 4 represents PAC between alpha and beta frequency bands in six channels without SL and over [−3, 2] s for all groups, which covers movement preparation and execution. The most notable differences are observable in PD + sFOG, which represents PAC between all alpha frequency sub-bands, including high alpha and low beta in Fz and Fc1. Similar PAC is observable in PD + mFOG in Fc1. In Figure 4, red dotted squares represent abnormal PAC between alpha and beta in PD with FOG groups. It should be noted that PAC between alpha and beta frequency bands was investigated over two different time intervals: [−3, 1] s and [−3, 2] s. High PAC between high alpha and beta was more pronounced over the latter time interval. Also, same time interval, [−3, 2] s, was investigated when SL is applied, and the results show the abnormal PAC between the alpha and beta frequency bands in PD + sFOG is no longer consistently high at the superficial layers.

## Discussion

Current knowledge of brain oscillation dynamics associated with FOG is very limited and does not reflect an integrative view toward this phenomenon (Snijders et al., 2016). Excessive theta and beta power has been repeatedly reported as FOG-related cortical abnormalities. In this study, we explored PLVs of eight frequency bands between 0.05 Hz and 35 Hz in HC and PD without and with different FOG severities. Two different spatial resolutions were investigated by utilizing a spatial filter, SL, to determine the abnormal phase synchronizations associated with FOG over superficial and deeper neural networks. Since the phase of the lower frequencies controls the amplitude of higher frequencies in neural oscillation and considering the fact that there are abnormal activities in the amplitude of some frequency bands, we also investigated the coupling between phase and amplitude of different frequency bands in case of observed abnormal phase synchrony associated with FOG. We found significant differences between phase features of PD with and without FOG over different cortical levels and frequency bands.

The remarkable phase-related abnormalities associated with FOG, and PD, can be summarized as: At superficial networks: (1) frontoparietal theta (left parietal areas) is observable only in PD + FOG groups. At deeper networks: (2) frontoparietal alpha band phase synchrony was shown to be associated with FOG and its severity, especially high alpha frequency band phase synchrony. (3) Alpha phase synchrony is observable mainly on the right parietal areas in PD with FOG groups, with exaggerated phase synchrony in PD + sFOG (4) Low beta phase synchrony is missing in PD + FOG. Abnormalities reported in PLV and PAC in PD patients with FOG may represent a chain of FOG-related features in phase and amplitude that can be interpreted in a meaningful way. As the low-frequency phase controls the high-frequency amplitude, abnormal PAC between theta and beta as well as alpha and beta frequency bands in PD patients with FOG emphasizes the possible role of the phase of theta and alpha in FOG. This means that abnormal reported frontoparietal theta, and alpha phase synchrony in PD with FOG might contribute to the amplitude abnormalities in higher frequencies such as beta, which is repetitively reported in the literature.

Brain oscillations coordinate various operations within and across neural networks in a timely manner (Buzsáki, 2009). Disturbed brain oscillations and phase synchronies thus indicate uncoordinated networks. Current results suggest that significantly high frontoparietal alpha sub-bands phase synchrony, especially high alpha, replaced the beta phase synchrony in deeper networks in PD patients with FOG. This feature was the most remarkable FOG-related feature. The mechanistic role of alpha oscillations in brain function as well as their generating structures are not entirely clear (Sadaghiani and Kleinschmidt, 2016). There is evidence that alpha cortical sources are located in deeper layers (layer V) of the occipital cortex, posterior regions (Knyazev, 2007), and could mediate feedback throughout the thalamocortical system. Slower alpha components are mainly generated in the anterior brain regions, while faster components are mostly located in the posterior regions (Knyazev, 2007). Aside from wide structures of alpha propagation, alpha oscillations are involved in multiple brain functions such as attention, memory, conscious perception, and sensory information integration (Chapeton et al., 2019). These functions are based on one of the main functions of alpha rhythm: cortical inhibition. In particular, phase-dependent alpha inhibition of cortical ongoing neural processing is a widely supported mechanism (Mathewson et al., 2011; Chapeton et al., 2019). Alpha phase determines the time and direction of a change in inhibition, and as a result, closely interacts with attentional blink implying that phase response reflects the attentional focus (Klimesch, 2012). On the other hand, attentional and working memory (WM) functions are thought to operate by similar underlying principles, and they often engage overlapping frontoparietal brain regions (Kiyonaga et al., 2021). Frontoparietal alpha-phase synchrony, in particular, reflects differential attentional demands during WM and visual attention (Kwon et al., 2015). Specifically, large-scale phase synchronization in frontoparietal network high alpha has been shown to be selectively associated with cognitive operations in visuospatial attention (Sadaghiani et al., 2012, 2019; Lobier et al., 2018). A recent study also suggests that alpha modulation creates a moment-by-moment trade-off between covert attention and WM (Klimesch, 2012; Van Moorselaar et al., 2022). In another study, a decrease in the anterior upper alpha oscillations was observed during central executive functions of WM (Sauseng et al., 2005). The results from the current study might therefore suggest unbalanced mechanisms between attention (especially visuospatial attention) and WM in PD patients with FOG. Furthermore, in the current study, alpha phase synchrony in the right hemisphere and left hemisphere showed different patterns in deeper networks (Figure 3). PD + FOG alpha phase synchronization between the right parietal hemisphere and the prefrontal area (Fz) is significantly higher than HC and PD-FOG. The bilateral parietal cortex has been shown to interact with WM and visual attention during dual-task demands. While the left parietal cortex strengthens the effect of WM content to guide attention toward matching visual targets, the right parietal cortex suppresses the effect of irrelevant visual distraction (Kiyonaga et al., 2021). As a result, reported higher upper alpha phase synchrony in PD + FOG and its association with FOG severity, especially on the right parietal areas at deeper networks, indicate that defective competition between WM with demands for subject’s attention in the environment could be considered as an underlying mechanism for FOG. This view is also consistent with previous studies that reported the association of visuospatial processing deficits and attention of the patient with FOG during walking (Nutt et al., 2011; Nantel et al., 2012; Silveira et al., 2015).

Frontoparietal theta phase synchrony at superficial networks and abnormal PAC involving theta frequency bands were other remarkable FOG-associated features. Prefrontal theta is phase-locked to hippocampal theta activity, and the frontoparietal theta is associated with visual WM (Knyazev, 2007; Kawasaki et al., 2014). Interestingly, a strong interplay between theta and posterior alpha phase has been reported in the literature (Sauseng et al., 2005; Williams et al., 2019). While posterior alpha activity is related to intuitive thinking and results in autonomic access to long-term memory, frontal theta activity is correlated with analytic thinking, which is reflective of cognitive control, WM, and attention (Williams et al., 2019). Short-term (episodic) memory demands lead to synchronization in the theta frequency band, whereas long-term (semantic) memory demands lead to a task-specific desynchronization in the upper alpha band (Knyazev, 2007). Theta frontoparietal coupling and a parallel decoupling of anterior regions in the upper alpha band have been introduced as plausible candidates for the neural correlates of the central executive function of WM (Sauseng et al., 2005). Consequently, this suggests that frontoparietal theta phase synchrony in more superficial networks might be a compensatory mechanism for higher alpha phase synchrony in the deeper neural pathways in PD + FOG. Moreover, high coupling between the phase of theta and the amplitude of low and high beta suggests the possible fundamental role of theta on abnormalities associated with FOG in the beta frequency band. The interplay between upper alpha and theta in WM relies on the prioritization of relevant information and suppression of irrelevant information. While prioritizing relevant information has been linked to theta frequency neural oscillations in the lateral PFC, suppressing irrelevant information has been linked to alpha oscillations in the occipito-parietal cortex (Riddle et al., 2020). Therefore, it can be concluded that in the case of PD + FOG, the impairment in visual attention in the deeper networks might be compensated by visual WM in more superficial networks as coherence in the frontoparietal network has been suggested to play a role in top-down control of spatial attention (Mathewson et al., 2014; van Schouwenburg et al., 2017). A recent study indicated that theta rhythms temporally resolve potential functional conflicts by periodically reweighting functional connections between higher-order brain regions and sensory or motor regions (Fiebelkorn and Kastner, 2019). Although gait is generally considered as an automatic movement, cortical control seems necessary to adapt gait patterns to environmental constraints (Delval et al., 2020). The abnormal frontoparietal high alpha and theta phase synchrony, along with an imbalance between left and right parietal alpha and theta phase synchrony, suggest the overemphasized visuospatial attention in PD with FOG patients. In other words, unsuppressed irrelevant visual distractions might trigger FOG events, which have been described previously while walking toward doorways, and also when cued to turn in the opposite direction (Almeida and Lebold, 2010; Lebold and Almeida, 2011; Knobl et al., 2012). When the temporoparietal cortex integrates visual, proprioceptive, and vestibular sensory information timely, the PM and SMA can generate the motor program accurately. This is especially true in an unfamiliar environment (Takakusaki, 2013; Gao et al., 2020). However, perceptual malfunction of visual inputs during locomotion planning might result in decreased speed and FOG episodes (Almeida and Lebold, 2010; Cowie et al., 2010, 2012; Cohen et al., 2011).

These findings are also consistent with structural abnormalities associated with FOG such as increased FOG with either left or bilateral stimulation and decreased by right STN stimulation and the fact that reduction of the gray matter in the left parietal lobe contributes to FOG in PD (Rubino et al., 2014; Mei et al., 2019; Pozzi et al., 2019). Consistent with the aforementioned points, PD + FOG showed significant dopaminergic deficits in the left caudate nucleus, which exhibited altered functional connectivity with regions of the visual network and possibly visual WM in deeper networks (Steidel et al., 2021). In HC and PD-FOG, alpha phase synchronization between parietal areas and M1 might therefore indicate effective connectivity between these areas resulting in a balance between attention and target task (Zazio et al., 2021).

A remarkable difference between PD groups without and with FOG was that PD-FOG represents similar distributions (frontoparietal PLV between Cp2- Fz, Cp2-Fc1, Cp1-Fc2), but over a different frequency band: low beta. PD + mFOG also represents similar frontoparietal interhemispheric patterns in the low beta frequency range at the superficial networks (with SL) between Cp2 and Fc2 as well as Cp2 and Fz, which corresponds to the low beta phase synchronizations in PD-FOG at deeper networks. Frontoparietal low beta phase synchrony in PD-FOG suggests similarity and fundamental differences between PD with and without FOG. Firstly, this pattern suggests that the frontoparietal phase synchrony in alpha vs. beta can be the identifier of the fundamental difference between PD with and without FOG at deeper layers. Since large-scale phase synchronization of brain rhythms has been suggested as a main concept in neural processes underlying cognition (Varela et al., 2001; Wang, 2010), low beta frequency band phase synchrony in frontoparietal networks in PD-FOG might indicate the exclusive neural activities and information transmission only about the task on hand, representing limited attention (Antzoulatos and Miller, 2016; D’Andrea et al., 2019; Gelastopoulos et al., 2019). Regarding the functional role of low beta, very little was found in the literature on low beta synchrony. However, low beta oscillation of the PFC was suggested to provide a substrate for an episodic buffer for WM, allowing a combination of executive commands (e.g., from PFC) and multimodal information into a flexible and updatable representation of recent sensory inputs (Gelastopoulos et al., 2019). The introduced alpha and beta interplay in PD with and without FOG might thus shed some light on the underlying mechanisms of the disease that lead to different symptoms in the patients.

Exaggerated low-beta power in global as well as local oscillatory synchronies in the beta frequency band within BG-Thalamo-Cortical network is a hallmark of PD pathophysiology (Choi et al., 2020), especially in PD with FOG. There is also evidence that there is a significant correlation between alpha and beta power spectrum and L-dopa intake, implying the role of dopaminergic mechanisms in the modulation of alpha and beta oscillations (Melgari et al., 2014). Low beta has been introduced as a locomotion-related feature in the mesencephalic locomotor region (MLR) (Noga et al., 2017). Lack of beta phase synchrony in the PD + FOG in deeper networks is consistent with the recent findings that suggest that FOG emerges when altered cortical control of gait is combined with a limited ability of the MLR to react to that alteration (Snijders et al., 2011), which might be due to environmental changes or visual attention. The increased beta amplitude may also indicate that the frontal generated motor plans failed to reach the motor cortex, resulting in the FOG events (Marquez et al., 2020). As a consequence, coupling of the phase of theta and high alpha with low beta amplitude in PD + FOG could be considered as a compensatory mechanism for the lack of beta phase synchrony in the deeper networks.

These findings provide key new information for the field’s basic understanding of underlying mechanisms of FOG. The results of the study suggest that the breakdown during FOG in the frontal lobe-BG-thalamo-cerebellar-brainstem network, which controls gait (Browner and Giladi, 2010), could be a result of not properly adapting to environmental stimuli. Moreover, drawn from the results and their interpretation, the large-scale frontoparietal phase synchrony of theta, alpha, and beta frequency bands along with the PAC between these frequency bands may be a useful biomarker of the severity of FOG, as well as a differential biomarker for PD with and without FOG. The phase of the brain oscillations provides a reliable yet non-invasive tool to investigate connectivity between various brain regions that can open a new window toward possibly a unified mechanism for FOG development and occurrence. However, it should be noted that, in the current study, the reference electrode was located on the soft tissue of the earlobe, contamination of which may cause distortions in PLVs. Although reference-free PLVs are also provided after applying SL, further research with other types of common reference EEG is required to confirm the PLV findings related to common reference EEG in this study.

Besides, the investigated frequency range was limited to 0.05–35 Hz in this study. The phase of the ultra-slow oscillation (<0.05 Hz) affects the slow oscillations such as theta and alpha and possibly higher oscillations (Buzsáki, 2009). So, it is crucial to investigate the association between the phase features of ultra-slow cortical potentials and FOG. Moreover, lack of left parietal low alpha phase synchrony at superficial layers might be related to the auditory cue during the movement task in the current protocol (Meyer et al., 2013; Deng et al., 2019). Further research should be undertaken to investigate the role of the phase of the left parietal low alpha frequency band in FOG. In addition, current study involved only cue-based movement execution tasks, further studies involving self-paced movement can help with developing a full picture of brain oscillations phase synchrony associated with FOG. Studies with more focus on phase synchrony patterns in multiple FOG subgroups, including cognitive, limbic, and motor subtypes can also help clarify the role of each frequency band phase synchronization in FOG. In addition, as previously mentioned, the frontoparietal theta is considered to be associated with visual WM. On the other hand, prefrontal theta is phase-locked to hippocampal theta activity (Knyazev, 2007; Kawasaki et al., 2014). The amplitude of theta also varies with the temporal evolution of FOG episode. Despite the importance of this frequency band in FOG, to the best of authors knowledge phase feature of theta has not been investigated in FOG in subcortical regions (Shine et al., 2013). Considering the coupling between the phase of theta and the amplitude of beta, it is worthwhile to investigate the phase of theta at both cortical and subcortical layers before or during FOG episodes.

## Data availability statement

The datasets presented in this study can be found in online repositories. The names of the repository/repositories and accession number(s) can be found below: http://ieee-dataport.org/documents/fog-severity-eegemg (Karimi, 2021).

## Ethics statement

The studies involving human participants were reviewed and approved by the Research Ethics Board at the University of Waterloo and Wilfrid Laurier University. The patients/participants provided their written informed consent to participate in this study.

## Author contributions

FK, NJ, and QA conceived and planned the experiments. FK carried out the experiments, developed the theoretical framework, analyzed the data, interpreted the results, and wrote the manuscript with input from all authors. QA contributed with medical expertise. NJ supervised the project. All authors reviewed and contributed to writing the manuscript.
